# Investigation of the Effects of Laser Welding Process Parameters on Weld Forming Quality Based on Orthogonal Experimental Design and Image Processing

**DOI:** 10.3390/ma18153627

**Published:** 2025-08-01

**Authors:** Yuewei Ai, Ning Sun, Shibo Han, Yang Zhang, Chang Lei

**Affiliations:** 1 School of Traffic and Transportation Engineering, Central South University, Changsha 410075, China; 2 Key Laboratory of Traffic Safety on Track of Ministry of Education, Central South University, Changsha 410075, China

**Keywords:** image processing, welding process parameters, weld forming quality, orthogonal experimental design, laser welding

## Abstract

Image processing has been widely adopted as an effective technology for analyzing weld forming quality which is greatly affected by the welding process parameters. In this paper, an *L*_25_(5^3^) orthogonal experiment is designed to investigate the effects of welding process parameters on the weld forming quality in laser welding of aluminum alloy. The weld characteristics including the weld width (*WW*), weld penetration (*PD*), weld area (*WA*) and weld porosity (*WP*) under the conditions of the different welding process parameters consisting of the laser power (*LP*), welding speed (*WS*) and defocus distance (*DD*) are extracted from the laser welding experiment based on image processing. The effectiveness of the weld characteristics extraction method is verified by comparing the extracted results with the measured results. It is found that the *WW*, *PD* and *WA* are all significantly influenced by the *LP* among the three welding process parameters while the influences of the three process parameters on the *WP* are insignificant. The *DD* has a significant influence on the *PD* and the *WS* has a significant influence on the *WA*. The corresponding significance of influence is lower than the significance of influence of *LP*. The analysis results are conducive to the optimization of laser welding process parameters and improvement of welding quality.

## 1. Introduction

Laser welding is as an ideal method to achieve the joining of aluminum alloy [1]. The weld porosity (*WP*) is a typical defect generated in aluminum alloy laser welding and the weld morphology characteristics are usually treated as the evaluation indices for welding quality [2,3,4]. The welding process parameters are the main factors affecting the welding quality. Therefore, investigating the effects of welding process parameters on the weld morphology characteristics and porosity is of great importance for optimizing the welding process parameters and improving the welding quality during laser welding of aluminum alloy.

With the development in computer technology, image processing has been widely applied to the field of laser welding for analyzing the welding process. Gao et al. [5] constructed a coaxial visual sensing system for laser welding process of stainless steel. Images of the molten pool and keyhole during the welding process were captured and denoised by Gaussian filter. The edges of the molten pool and keyhole under the conditions of the different welding process parameters were obtained. Lei et al. [6] collected the molten pool images in laser welding process of Ti6Al4V alloy. Grayscale processing and binary processing were used to acquire the contour of the keyhole and then obtain the mean diameter of the keyhole. Liu et al. [7] extracted the top keyhole area based on the keyhole image from the top view and acquired the keyhole depth based on the keyhole image from the lateral view. The keyhole boundary profile was extracted using morphological gradient techniques. Huang et al. [8] examined the influence of magnesium content on the keyhole behavior during laser welding of 5083, 5754 and 5A06 Al-Mg alloys. Characteristics of the keyhole area were extracted from the high-speed image. The small noise was removed from the images by the Gaussian filter and the redundant parts were removed by the erosion and dilation operations to obtain a smooth keyhole shape. Nguyen and Lee [9] developed a laser-vision-based weld quality inspection system. The laser stripe produced by the intersection of the laser plane and the weld surface was captured by the camera. Based on the feature points on the extracted laser stripe of the weld profile image, the weld dimensions were measured.

To meet the service requirement of components with aluminum alloy welded joints, the optimization of laser welding process parameters has been widely investigated. Adisa et al. [10] analyzed laser welding process of aluminum alloy under the conditions of the different welding process parameters. It was found that the welding speed (*WS*) and peak power had great influences on the aluminum alloy weldability. The largest weld depth-width ratio was obtained under the condition of the *WS* 1 mm/s and peak power 0.91 kW. Liu et al. [11] investigated the effect of oscillation amplitude on the *WP* in circular oscillation laser welding of medium-thick high-magnesium aluminum alloy by the control variate method. They found that the *WP* could be reduced from 11.2% to 5.2% as the oscillation amplitude increasing from 0 mm to 1 mm. Casalino et al. [12] proposed an artificial neural network to investigate the effects of welding process parameters on welding quality of AA5754 aluminum alloy. The weld morphology characteristics obtained by the artificial neural network were compared with the experimental results and they found that the mean absolute percentage error was less than 4%. Wang et al. [13] acquired the weld geometric morphology data in laser-TIG welding experiments of 6061 aluminum alloy. In their research, the back propagation neural network optimized by the genetic algorithm was adopted to predict the weld geometric morphology data. The results showed that the average accuracy of the prediction model could reach 97%. Wu et al. [14] established the relation model between the welding process parameters and weld morphologies. The non-dominated sorting genetic algorithm-II was used to explore the Pareto optimal solutions of the welding process parameters and the obtained optimal results agreed well with the experimental results. Zhang et al. [15] introduced a circular oscillating laser to guide the regular flow of molten pool. An optimal laser oscillating welding was developed to effectively control porosity within the acceptable range using response surface methodology.

From the above analysis, image processing is a powerful technology to analyze the weld morphology and porosity which are mainly affected by the welding process parameters in laser welding of aluminum alloy. Therefore, the effects of the laser power (*LP*), *WS* and defocus distance (*DD*) on the weld width (*WW*), weld penetration (*PD*), weld area (*WA*) and *WP* are investigated in this paper based on the orthogonal experimental design and image processing. The results can provide the theoretical guidance for optimizing welding process parameters in laser welding of aluminum alloy, which is helpful for improving the weld morphology.

## 2. Experimental Procedure

### 2.1. Material

The 6061 aluminum alloy is selected as the base material in this research. The dimension of the welding specimens is 220 mm × 100 mm × 2 mm. Since the aluminum alloy is easily oxidized, the surfaces of the welding specimens are covered with oxide film which promotes the formation of weld defects during laser welding process. The surfaces of the welding specimens are polished by the sandpaper and cleaned by the ethanol solution to remove the oxide film and impurities before the welding experiment.

### 2.2. Orthogonal Experimental Design

To investigate the effects of laser welding process parameters on weld forming quality, the experiment is designed based on an *L*_25_(5^3^) orthogonal table [16]. The three factors are *LP*, *WS* and *DD*. The factors and corresponding levels in the orthogonal experiment are tabulated in Table 1. The orthogonal table used in this research is as shown in Table 2.

### 2.3. Welding System

**Figure 1:**
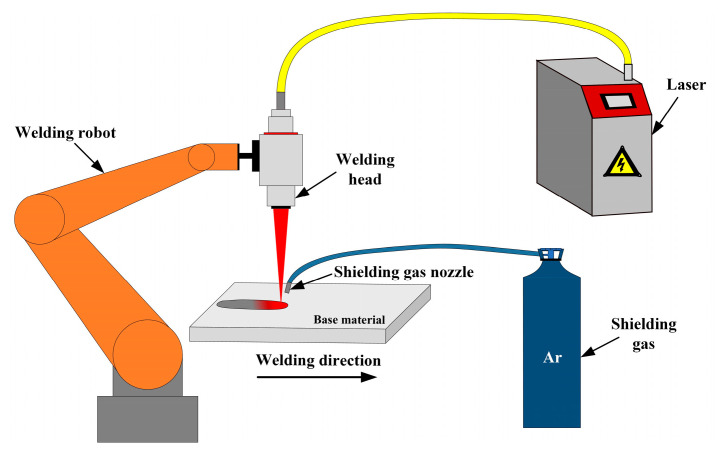
Schematic diagram of the welding system.

The laser welding processes of aluminum alloy under the conditions of the different welding process parameters are achieved by the welding system. The welding system is composed of the laser, welding head, welding robot, shielding gas nozzle and so on. The wavelength of the laser is 1064 nm and the welding head is fixed on the welding robot. During laser welding process, the argon gas with the flow rate of 20 L/min is used as the shielding gas to protect the welding zone. The schematic diagram of the welding system is shown in Figure 1.

Weld samples are cut from the zones close to the weld centerline and then placed inside the metallographic sample inlay equipment. The metallographic samples are polished with sandpaper and polishing agent after inlaying. Then, the metallographic samples are etched to improve the weld zones contrast. The metallographic images of weld are obtained by the optical microscope.

## 3. Weld Characteristics Extraction

### 3.1. Weld Morphology Characteristics Extraction

**Figure 2:**
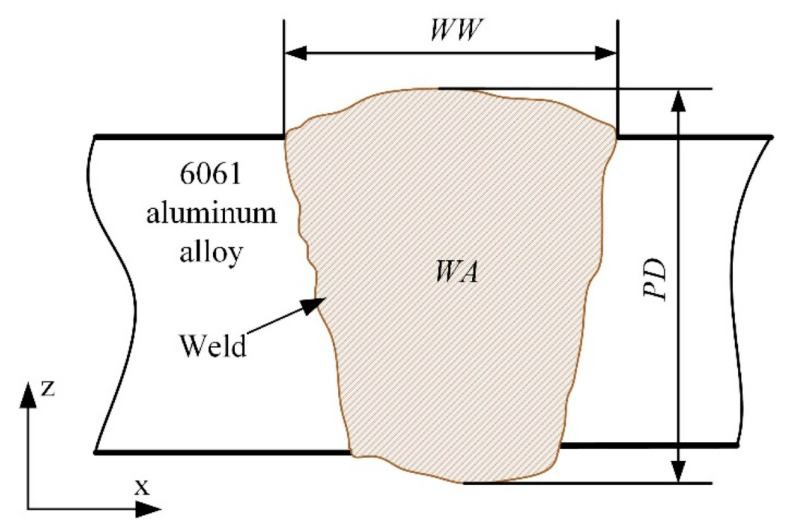
Schematic diagram of weld morphology characteristics.

In this paper, the *WW*, *PD* and *WA* are selected as the weld morphology characteristics which are extracted from the metallographic images of weld. The schematic diagram of weld morphology characteristics is shown in Figure 2.

Based on the traditional extraction method, the weld morphology characteristics including the *WW* and *PD* are obtained by importing the metallographic image of the weld into the measurement software and measuring. This method is inefficient when processing a large number of metallographic images. Additionally, it is difficult to obtain the *WA* directly for the weld with irregular shape. Therefore, image processing is adopted in the current research to extract the weld morphology characteristics.

**Figure 3:**
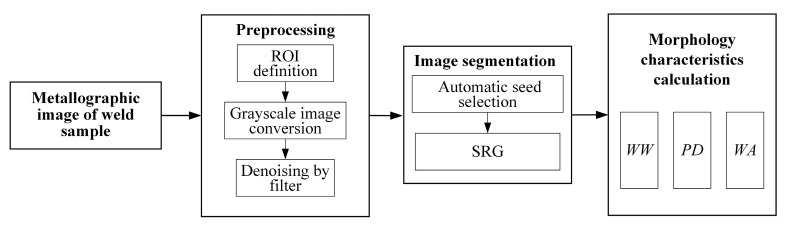
Flow chart of weld morphology characteristics extraction.

The flow chart of weld morphology characteristics extraction is shown in Figure 3. The metallographic image of the weld is preprocessed through region of interest (ROI) definition, grayscale image conversion and denoising by filter. Then, the automatic seed selection is performed and the seeded region growing (SRG) algorithm [17] is applied for segmentation. Based on the processed image, the weld morphology characteristics including the *WW*, *PD* and *WA* are calculated.

To reduce computational load and noise interference, the region close to the weld is designated as the ROI as follows [18]:

(1) $$\left\{\begin{array}{l} x_{A}=x_{D}=x_{0}\\ z_{A}=z_{B}=z_{0}\\ x_{C}=x_{B}=x_{0}+w\\ z_{C}=z_{D}=z_{0}+h \end{array}\right.$$

where points *A*, *B*, *C* and *D* are the bottom-left point, bottom-right point, top-right point and top-left point of the ROI, respectively. $x_{A}$, $x_{B}$, $x_{C}$ and $x_{D}$ are the x coordinates of points *A*, *B*, *C* and *D*, respectively. $z_{A}$, $z_{B}$, $z_{C}$ and $z_{D}$ are the z coordinates of points *A*, *B*, *C* and *D*, respectively. $x_{0}$ and $z_{0}$ are set according to the metallographic image of weld sample. *w* is the width of the ROI and *h* is the height of the ROI.

**Figure 4:**
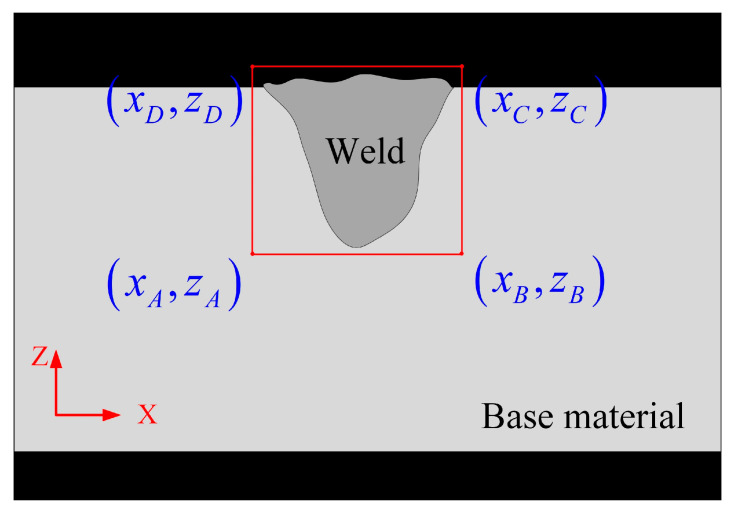
Schematic diagram of ROI extraction.

The ROI is a rectangular region defined by these four points as shown in Figure 4, which allows for precise segmentation and analysis of specific area within the metallographic image of the weld.

During the grayscale image conversion, the colorful metallographic image of the weld in RGB system is converted to grayscale image. The gray level $g_{\left(i,j\right)}$ of point (i,j) in the grayscale image is the weighted sum of the red, green and blue channel values of the original image, which can be defined as [19]:

(2) $$g_{\left(i,j\right)}=0.299\times R_{\left(i,j\right)}+0.587\times G_{\left(i,j\right)}+0.114\times B_{\left(i,j\right)}$$

where $R_{\left(i,j\right)}$, $G_{\left(i,j\right)}$ and $B_{\left(i,j\right)}$ are the red, green and blue channel values of point (i,j), respectively. The value ranges of the red, green and blue channel values are 0–255.

The noise points in the image have great effect on the segmentation result. In the current research, the mean filter is used to denoise the grayscale image. The filtering process is to convert the gray level of a pixel into the mean value of the gray level of the pixel matrix where the pixel is located in the center. The filtering process is described as [20]:

(3) $$g_{m(i,j)}=\frac{1}{n\times n}\sum_{(i,j)\in C_{m}}g_{\left(i,j\right)}$$

where $g_{m(i,j)}$ is gray level of point (i,j) after filtering process. $C_{m}$ is a n×n pixel matrix where the point (i,j) is located in the center.

The image segmentation process including the automatic selection of the initial seed and the SRG algorithm can be referenced from Ref. [17]. Based on the image segmentation result, the weld morphology characteristics including the *WW*, *PD* and *WA* are calculated as follows [21]:

(4) $$WW=\left|x_{max}-x_{min}\right|\times r_{con}$$

(5) $$PD=\left|z_{max}-z_{min}\right|\times r_{con}$$

(6) $$WA=P_{w}\times r_{con}^{2}$$

where $x_{min}$, $x_{max}$, $z_{min}$ and $z_{max}$ are the minimum x coordinate, maximum x coordinate, minimum z coordinate and maximum z coordinate of the image segmentation result, respectively. $P_{w}$ is the number of pixels located in the weld zone. $r_{con}$ is the actual length of one pixel in the metallographic image of weld.

### 3.2. WP Extraction

The weld pore defect is a typical defect in laser welding of aluminum alloy. Based on the preprocessed image, the binary processing is carried out according to the gray level range of the pore region as follows [22]:

(7) $$g_{b(i,j)}=\left\{\begin{array}{cc} 255 & g_{\left(i,j\right)}\in R_{p}\\ 0 & g_{\left(i,j\right)}\notin R_{p} \end{array}\right.$$

where $g_{b(i,j)}$ is the gray level of point (i,j) after binary processing. $R_{p}$ is the gray level range of pore region.

The *WP* is calculated as [23]:

(8) $$WP=\frac{P_{p}}{P_{w}}\times 100\%$$

where $P_{p}$ is the number of pixels located in the pore region.

## 4. Results and Discussion

### 4.1. Weld Morphology Characteristics Extraction Results

**Figure 5:**
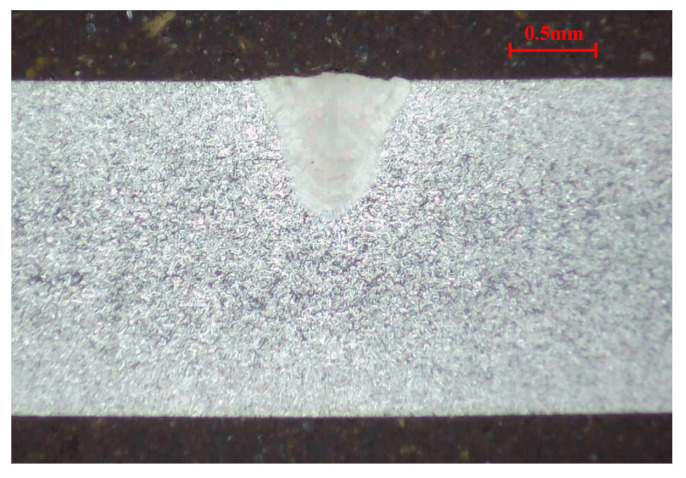
Cross-section metallographic image of the weld sample from the No. 4 group of the experiment.

Based on the weld morphology characteristics extraction process, the weld morphology characteristics of twenty-five metallographic images of weld samples are extracted. Taking the cross-section metallographic image of the weld sample from the No. 4 group of the experiment (*LP* 700 W, *WS* 4.0 m/min and *DD* +1.0 mm) as an example, the corresponding metallographic image of the weld is shown in Figure 5. The corresponding $r_{con}$ in Figure 5 is 0.0033 mm.

**Figure 6:**
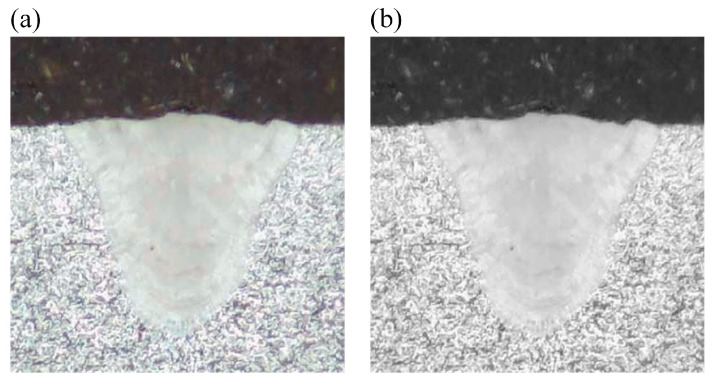
ROI image and grayscale image: (**a**) ROI image; (**b**) grayscale image.

**Figure 7:**
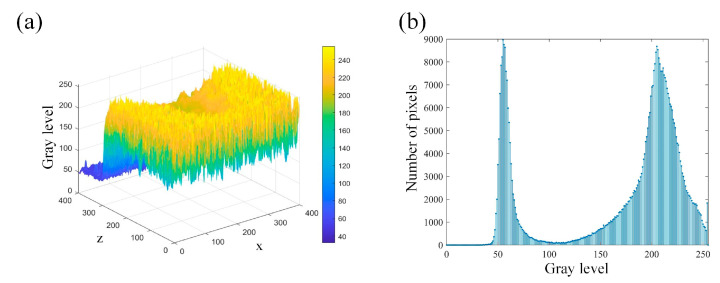
Gray levels distribution and gray histogram: (**a**) gray levels distribution; (**b**) gray histogram.

**Figure 8:**
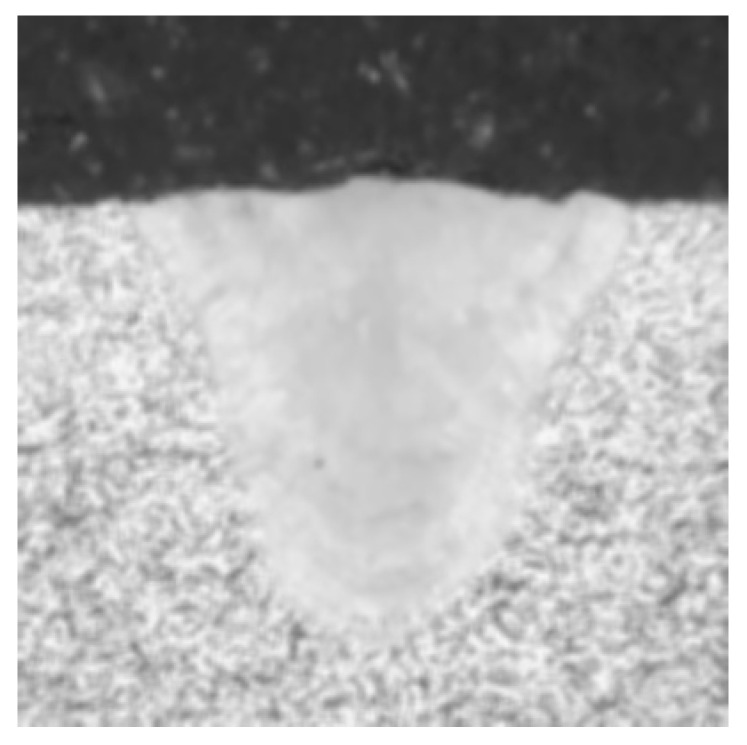
Grayscale image after filtering process.

For the selected metallographic image, the preprocessing is conducted. The weld is selected as the center region of ROI and both of the *w* and *h* are set as 400. The obtained ROI image is shown in Figure 6a. After the grayscale image conversion, the corresponding grayscale image is shown in Figure 6b. From Figure 7a, it is clearly observed that the gray levels of weld zone are mainly distributed in the range of 160–220, and the gray levels of base material zone are mainly distributed in the range of 220–250. From Figure 7b, most of the gray levels of pixels are concentrated in the ranges of 50–100 and 150–250. According to Figure 7, the gray levels range of 50–100 correspond to the inlay material zone and the gray levels range of 150–250 correspond to the weld and base material zone. The grayscale image after filtering process is shown in Figure 8.

**Figure 9:**
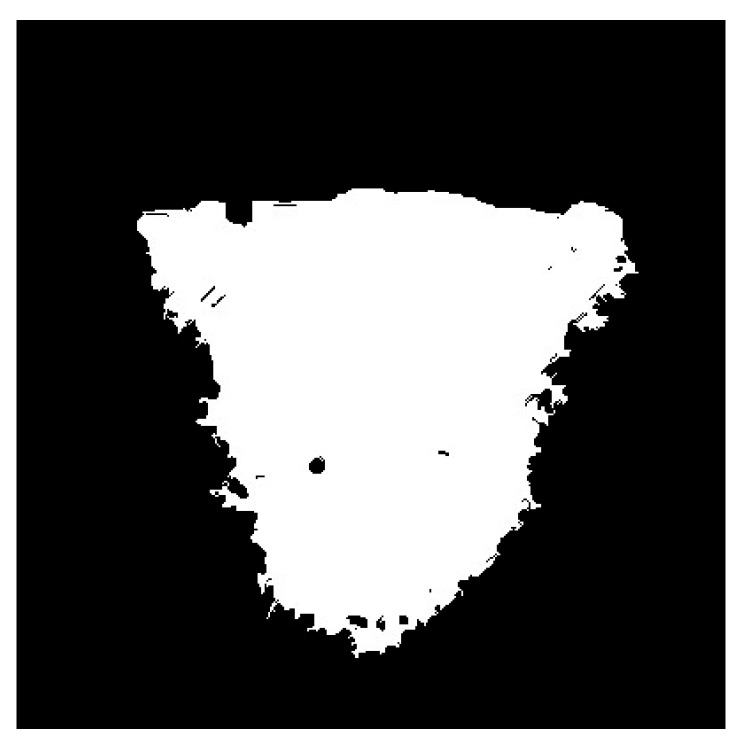
Image segmentation result.

After the preprocessing of the metallographic image of weld, the SRG algorithm with the automatic initial seed selection is applied to segment the grayscale image after filtering process. The image segmentation result is shown in Figure 9.

**Figure 10:**
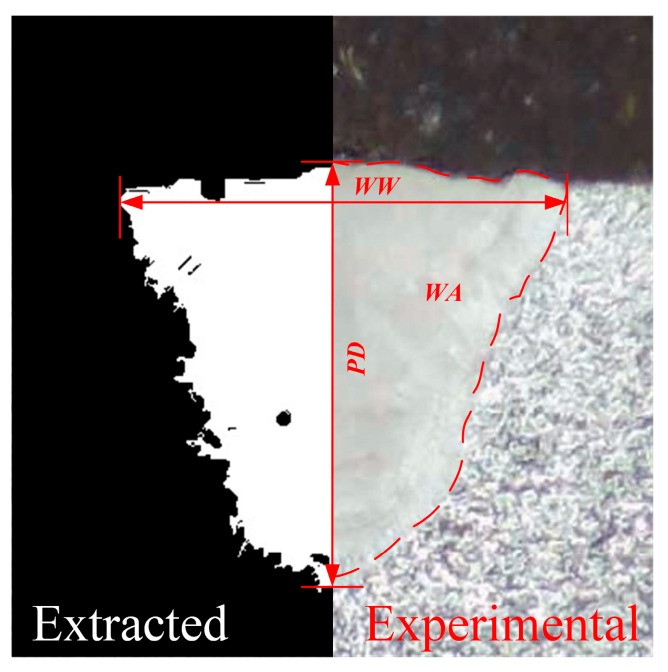
Comparison between the extracted weld morphology and the experimental weld morphology.

The extracted weld morphology is compared with the experimental weld morphology, as shown in Figure 10. It can be found that the extracted weld morphology agree well with the experimental weld morphology. The extracted weld morphology characteristics of the twenty-five weld samples are shown in Table 3.

As shown in Table 3, five weld samples are randomly selected to validate the effectiveness of the proposed method for extracting weld morphology characteristics. After manually annotating the weld areas to be measured, the marked images are imported into the measurement software for measuring. The corresponding extracted weld morphology characteristics are compared with the measured weld morphology characteristics by the measurement software, as shown in Table 4. It can be clearly seen that all relative errors are less than 5%, which demonstrates the effectiveness of the proposed method for extracting weld morphology characteristics.

### 4.2. WP Extraction Results

**Figure 11:**
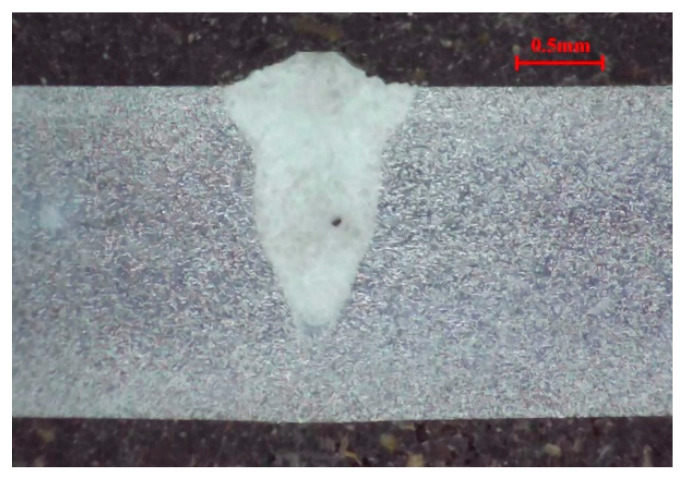
Cross-section metallographic image of the weld sample from the No. 9 group of the experiment.

**Figure 12:**
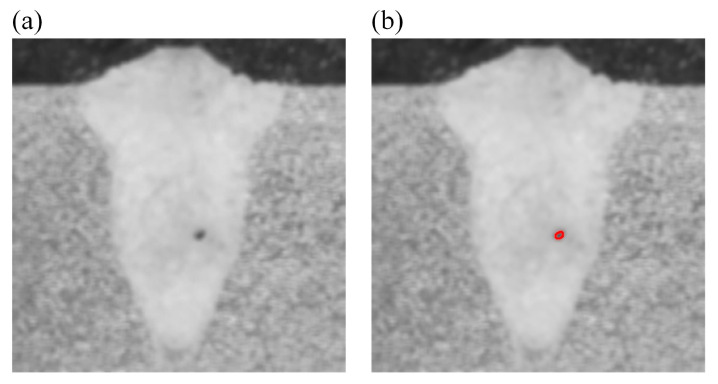
Image after preprocessing and pore profile extraction result: (**a**) image after preprocessing; (**b**) pore profile extraction result.

Based on the *WP* extraction process, the *WP*s of twenty-five metallographic images of weld samples are extracted. Taking the cross-section metallographic image of the weld sample from the No. 9 group of the experiment (*LP* 900 W, *WS* 4.0 m/min and *DD* +2.0 mm) as an example, the corresponding metallographic image of the weld is shown in Figure 11. The image after preprocessing is shown in Figure 12a. According to the gray level range of the image after preprocessing, the binary processing is carried out and the obtained pore profile extraction result is shown in Figure 12b. The extracted *WP*s of the twenty-five weld samples are shown in Table 5.

### 4.3. Effects of Welding Process Parameters on Weld Morphology

**Figure 13:**
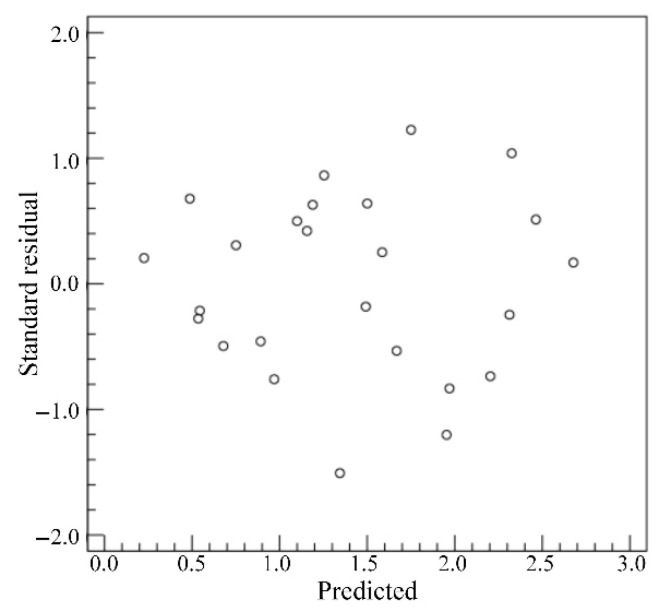
Standard residuals for *WW*.

**Figure 14:**
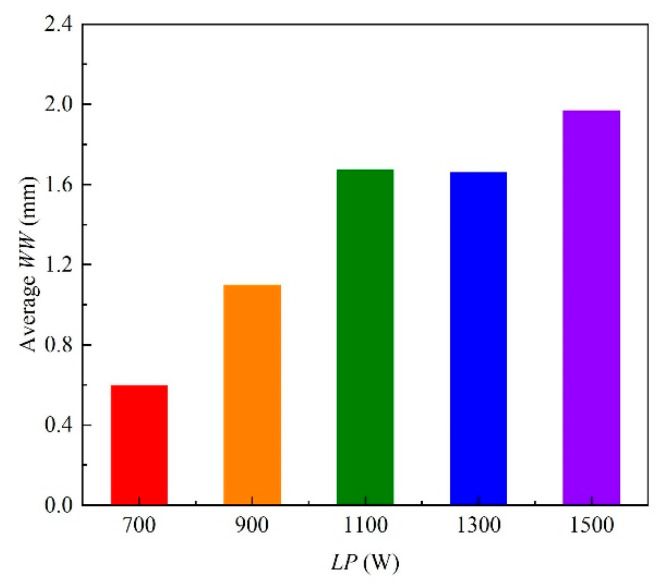
Relationship between the average *WW* and the levels of *LP*.

Based on the extracted weld morphology characteristics of twenty-five weld samples, the weld morphology characteristics under the conditions of the different welding process parameters are analyzed. For the *WW*, the standard residuals are calculated when the dependent variable is *WW*, as shown in Figure 13. From Figure 13, the standard residuals are relatively uniformly distributed on both sides of 0, which indicates that the data meet the homogeneity of variance and can be used for multivariate analysis of variance. By setting the *WW* as the dependent variable and the *LP*, *WS* and *DD* as the impact factors, the test of intersubjective effects is conducted and the corresponding results are as shown in Table 6. From Table 6, the *p* value of *LP* is less than 0.05, which indicates that the *LP* has a significant influence on the *WW*. The relationship between the average *WW* and the levels of *LP* is shown in Figure 14. The average *WW* is the average value of the *WW*s of a factor with a certain level. From Figure 14, it can be seen that the average *WW* generally increases with the increase in *LP*.

**Figure 15:**
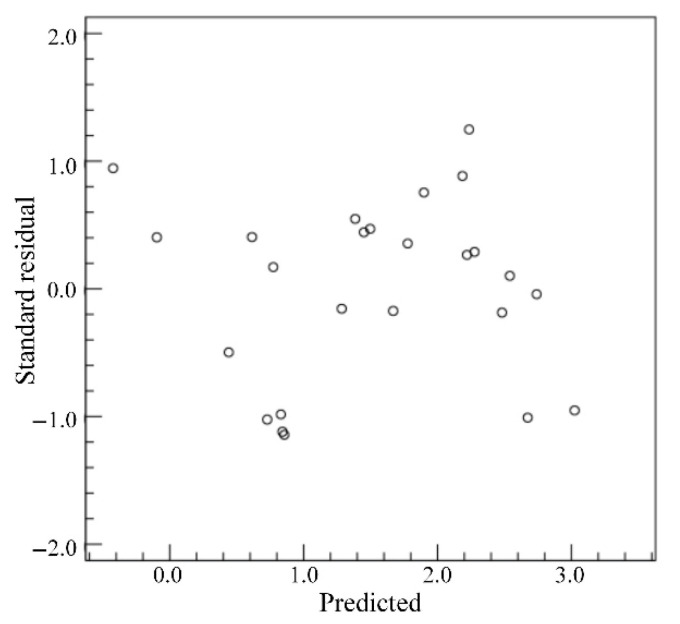
Standard residuals for *PD*.

**Figure 16:**
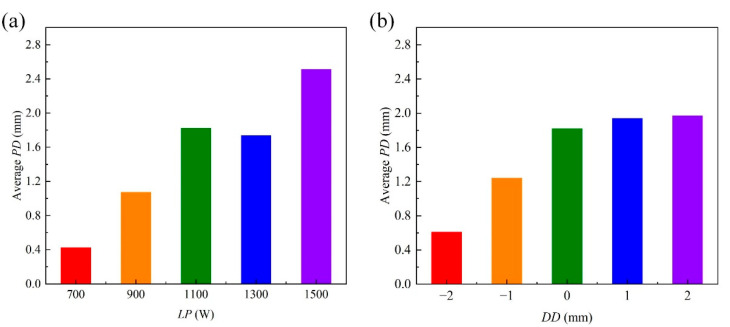
Relationships between the average *PD* and the levels of factors: (**a**) *LP*; (**b**) *DD*.

For the *PD*, the standard residuals are calculated when the dependent variable is *PD*, as shown in Figure 15. From Figure 15, the standard residuals are relatively uniformly distributed on both sides of 0, which indicates that the data meet the homogeneity of variance and can be used for multivariate analysis of variance. By setting the *PD* as the dependent variable and the *LP*, *WS* and *DD* as the impact factors, the test of intersubjective effects is conducted and the corresponding results are as shown in Table 7. From Table 7, the *p* values of *LP* and *DD* are less than 0.05, which indicates that the *LP* and *DD* have significant influences on the *PD*. Therefore, only the correlation analyses of *LP* and *DD* on the *PD* are conducted. The relationships between the average *PD* and the levels of factors are shown in Figure 16. From Figure 16, the average *PD* generally increases with the increase in *LP* and increases with the increase in *DD*. The difference between the maximum and minimum values in Figure 16a is larger than that in Figure 16b, which indicates that the significance of the influence of *LP* on the *PD* is higher than the significance of the influence of *DD* on the *PD.*

**Figure 17:**
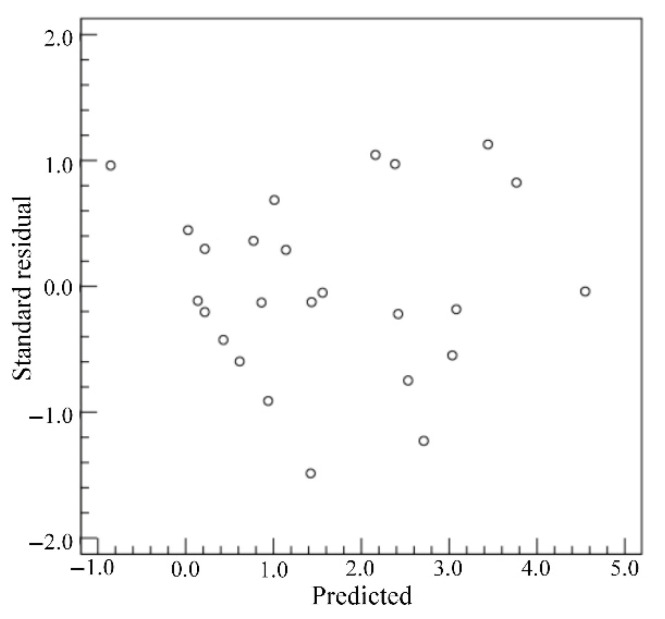
Standard residuals for *WA*.

**Figure 18:**
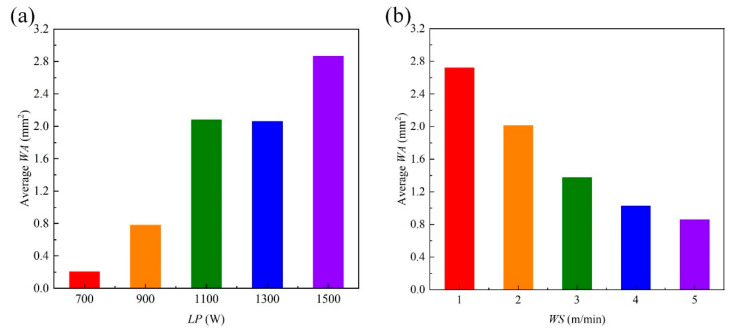
Relationships between the average *WA* and the levels of factors: (**a**) *LP*; (**b**) *WS*.

For the *WA*, the standard residuals are calculated when the dependent variable is *WA*, as shown in Figure 17. From Figure 17, the standard residuals are relatively uniformly distributed on both sides of 0, which indicates that the data meet the homogeneity of variance and can be used for multivariate analysis of variance. By setting the *WA* as the dependent variable and the *LP*, *WS* and *DD* as the impact factors, the test of intersubjective effects is conducted and the corresponding results are shown in Table 8. From Table 8, the *p* values of *LP* and *WS* are less than 0.05, which indicates that the *LP* and *WS* have significant influences on the *WA*. The relationships between the average *WA* and the levels of factors are as shown in Figure 18. From Figure 18, the average *WA* generally increases with the increase in *LP* and decreases with the increase in *WS*. The difference between the maximum and minimum values in Figure 18a is larger than that in Figure 18b, which indicates that the significance of the influence of *LP* on the *WA* is higher than the significance of the influence of *WS* on the *WA*.

### 4.4. Effects of Welding Process Parameters on the WP

**Figure 19:**
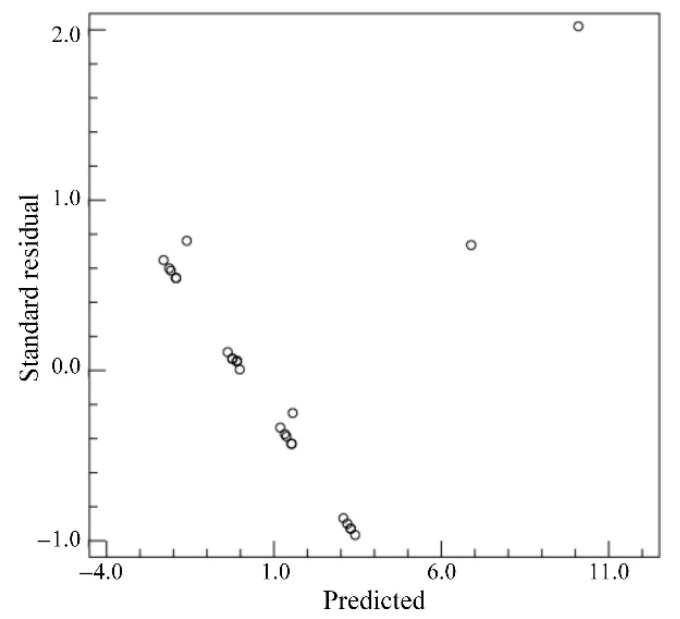
Standard residuals for *WP*.

Based on the extracted *WP*s of twenty-five weld samples, the *WP*s under the conditions of the different welding process parameters are analyzed. The standard residuals are calculated when the dependent variable is *WP*, as shown in Figure 19. From Figure 19, the maximum standard residual is 2.021 and the distribution of standard residuals is relatively concentrated, which indicates that the data of *WP*s have relatively strong randomness and may not meet the homogeneity of variance. By setting the *WP* as the dependent variable and the *LP*, *WS* and *DD* as the impact factors, the factor correlation analysis is conducted and the corresponding results are as shown in Table 9. From Table 9, the *p* values of *LP*, *WS* and *DD* are greater than 0.05, which indicates that the influences of *LP*, *WS* and *DD* on the *WP* are insignificant.

## 5. Conclusions

The influences of welding process parameters in aluminum alloy laser welding are explored in this study based on orthogonal experimental design and image processing. An *L*_25_(5^3^) orthogonal experiment is designed and the metallographic images of twenty-five weld samples are obtained from the experiment. The weld characteristics including the *WW*, *PD*, *WA* and *WP* under the conditions of the different welding process parameters considering the *LP*, *WS* and *DD* are extracted from the metallographic images by image processing. The extracted weld characteristics are selected randomly to compare with the measured weld characteristics and the effectiveness of the proposed method is validated. The results show that the *LP* has a significant influence on the *WW*. Both the *LP* and *DD* have significant influences on the *PD* and the influence significance of *LP* is higher. For the *WA*, the influences of the *LP* and *WS* are significant and the influence significance of *LP* is higher. Additionally, the data of *WP*s have relatively strong randomness and the influences of *LP*, *WS* and *DD* on the *WP* are insignificant. The proposed method is benefit for extracting the weld characteristics and the analysis results can provide the theoretical support for optimizing the laser welding parameters and improving the weld quality.

## Figures and Tables

**Table 1:** Factors and corresponding levels in orthogonal experiment.

Factor	Level 1	Level 2	Level 3	Level 4	Level 5
*LP* (W)	700	900	1100	1300	1500
*WS* (m/min)	1.0	2.0	3.0	4.0	5.0
*DD* (mm)	−2.0	−1.0	0.0	+1.0	+2.0

**Table 2:** The orthogonal table.

No.	*LP* (W)	*WS* (m/min)	*DD* (mm)	No.	*LP* (W)	*WS* (m/min)	*DD* (mm)
1	700	1.0	−2.0	14	1100	4.0	−2.0
2	700	2.0	−1.0	15	1100	5.0	−1.0
3	700	3.0	0.0	16	1300	1.0	+1.0
4	700	4.0	+1.0	17	1300	2.0	+2.0
5	700	5.0	+2.0	18	1300	3.0	−2.0
6	900	1.0	−1.0	19	1300	4.0	−1.0
7	900	2.0	0.0	20	1300	5.0	0.0
8	900	3.0	+1.0	21	1500	1.0	+2.0
9	900	4.0	+2.0	22	1500	2.0	−2.0
10	900	5.0	−2.0	23	1500	3.0	−1.0
11	1100	1.0	0.0	24	1500	4.0	0.0
12	1100	2.0	+1.0	25	1500	5.0	+1.0
13	1100	3.0	+2.0				

**Table 3:** Extracted weld morphology characteristics of the twenty-five weld samples.

No.	*WW* (mm)	*PD* (mm)	*WA* (mm^2^)	No.	*WW* (mm)	*PD* (mm)	*WA* (mm^2^)
1	0.417	0.136	0.036	14	0.517	0.179	0.063
2	0.384	0.146	0.032	15	1.397	1.192	0.744
3	0.371	0.122	0.029	16	2.768	2.377	4.528
4	0.934	0.874	0.493	17	1.765	2.371	2.528
5	0.891	0.854	0.440	18	0.619	0.248	0.097
6	0.447	0.179	0.048	19	1.768	1.709	1.643
7	1.735	1.566	1.317	20	1.384	1.987	1.512
8	1.563	1.775	1.409	21	2.778	2.460	4.511
9	1.407	1.712	1.106	22	2.480	2.344	3.282
10	0.348	0.142	0.034	23	1.881	2.974	3.127
11	2.944	2.709	4.485	24	1.474	2.715	1.840
12	2.166	2.600	2.912	25	1.238	2.076	1.575
13	1.351	2.447	2.216				

**Table 4:** Comparison between the extracted weld morphology characteristics and the measured weld morphology characteristics of the five weld samples.

No.	Extracted Weld Morphology Characteristics			Measured Weld Morphology Characteristics			Relative Errors		
	*WW* (mm)	*PD* (mm)	*WA* (mm^2^)	*WW* (mm)	*PD* (mm)	*WA* (mm^2^)	*WW* (%)	*PD* (%)	*WA* (%)
13	1.351	2.447	2.216	1.333	2.459	2.171	1.350	0.488	2.073
14	0.517	0.179	0.063	0.533	0.177	0.061	3.002	1.130	3.279
18	0.619	0.248	0.097	0.646	0.237	0.093	4.180	4.641	4.301
22	2.480	2.344	3.282	2.382	2.362	3.187	4.114	0.762	2.981
23	1.881	2.974	3.127	1.806	3.024	3.119	4.153	1.653	0.256

**Table 5:** Extracted *WP*s of the twenty-five weld samples.

No.	*WP* (%)	No.	*WP* (%)	No.	*WP* (%)	No.	*WP* (%)	No.	*WP* (%)
1	0.000	6	0.000	11	0.675	16	0.000	21	0.000
2	0.000	7	0.000	12	0.000	17	0.000	22	0.000
3	0.000	8	1.097	13	0.000	18	0.000	23	0.000
4	0.073	9	0.102	14	0.000	19	0.000	24	0.000
5	9.500	10	0.000	15	0.000	20	17.256	25	0.000

**Table 6:** Factor correlation analysis of *WW*.

Source	Sum of Squares	Degree of Freedom	Mean Square	F Value	*p* Value
Correction model	11.269	12	0.939	2.652	0.052
Intercept	49.076	1	49.076	138.586	0.000
*LP*	5.999	4	1.500	4.235	0.023
*WS*	2.641	4	0.660	1.864	0.182
*DD*	2.630	4	0.658	1.857	0.183
Error	4.249	12	0.354		
Total	64.594	25			
Total of corrections	15.519	24			

**Table 7:** Factor correlation analysis of *PD*.

Source	Sum of Squares	Degree of Freedom	Mean Square	F Value	*p* Value
Correction model	20.306	12	1.692	4.831	0.005
Intercept	57.438	1	57.438	163.990	0.000
*LP*	12.613	4	3.153	9.003	0.001
*WS*	0.818	4	0.205	0.584	0.680
*DD*	6.874	4	1.719	4.906	0.014
Error	4.203	12	0.350		
Total	81.947	25			
Total of corrections	24.509	24			

**Table 8:** Factor correlation analysis of *WA*.

Source	Sum of Squares	Degree of Freedom	Mean Square	F Value	*p* Value
Correction model	43.816	12	3.651	4.268	0.009
Intercept	64.022	1	64.022	74.844	0.000
*LP*	23.318	4	5.830	6.815	0.004
*WS*	11.760	4	2.940	3.437	0.043
*DD*	8.737	4	2.184	2.553	0.093
Error	10.265	12	0.855		
Total	118.103	25			
Total of corrections	54.081	24			

**Table 9:** Factor correlation analysis of *WP*.

Source	Sum of Squares	Degree of Freedom	Mean Square	F Value	*p* Value
Correction model	205.930	12	17.161	1.365	0.299
Intercept	32.954	1	32.954	2.622	0.131
*LP*	45.307	4	11.327	0.901	0.493
*WS*	110.560	4	27.640	2.199	0.131
*DD*	50.063	4	12.516	0.996	0.447
Error	150.810	12	12.568		
Total	389.694	25			
Total of corrections	356.740	24			

## Data Availability

The original contributions presented in this study are included in the article. Further inquiries can be directed to the corresponding author.

## References

[1] Cai J., Wei Y., Ouyang Z., Liu X., Jin H., Chen J. (2024). Investigation on clockwise circular oscillating laser welding for the 5A06-H112 aluminum alloy: Energy distribution, seam appearance, microstructure, and mechanical properties. Opt. Laser Technol..

[2] Xu G., Li P., Li L., Hu Q., Zhu J., Gu X., Du B. (2019). Influence of Arc Power on Keyhole-Induced Porosity in Laser plus GMAW Hybrid Welding of Aluminum Alloy: Numerical and Experimental Studies. Materials.

[3] Wang L., Yao M., Gao X., Kong F., Tang J., Kim M.J. (2023). Keyhole stability and surface quality during novel adjustable-ring mode laser (ARM) welding of aluminum alloy. Opt. Laser Technol..

[4] Kang S., Shin J. (2021). Laser beam oscillation welding of aluminum alloy using the spatially modulated beam by diffractive optical element (DOE). J. Manuf. Process..

[5] Gao J.-Q., Qin G.-L., Yang J.-L., He J.-G., Zhang T., Wu C.-S. (2011). Image processing of weld pool and keyhole in Nd:YAG laser welding of stainless steel based on visual sensing. Trans. Nonferrous Met. Soc. China.

[6] Lei Z., Shen J., Wang Q., Chen Y. (2019). Real-time weld geometry prediction based on multi-information using neural network optimized by PCA and GA during thin-plate laser welding. J. Manuf. Process..

[7] Liu S., Wu D., Luo Z., Zhang P., Ye X., Yu Z. (2022). Measurement of pulsed laser welding penetration based on keyhole dynamics and deep learning approach. Measurement.

[8] Huang Y., Shen C., Ji X., Li F., Zhang Y., Hua X. (2020). Effects of Mg content on keyhole behaviour during deep penetration laser welding of Al-Mg alloys. Opt. Laser Technol..

[9] Nguyen H.-C., Lee B.-R. (2014). Laser-Vision-based Quality Inspection System for Small-Bead Laser Welding. Int. J. Precis. Eng. Manuf..

[10] Adisa S.B., Loginova I., Khalil A., Solonin A. (2018). Effect of Laser Welding Process Parameters and Filler Metals on the Weldability and the Mechanical Properties of AA7020 Aluminium Alloy. J. Manuf. Mater. Process..

[11] Liu M., Shao C., Zheng Z., Lu F. (2024). The effect of laser oscillation welding on porosity suppression for medium-thick Al alloy with high Mg content. Opt. Laser Technol..

[12] Casalino G., Facchini F., Mortello M., Mummolo G. (2016). ANN modelling to optimize manufacturing processes: The case of laser welding. IFAC-PapersOnLine.

[13] Wang H., Zhang Z., Liu L. (2021). Prediction and fitting of weld morphology of Al alloy-CFRP welding-rivet hybrid bonding joint based on GA-BP neural network. J. Manuf. Process..

[14] Wu J., Zhang S., Sun J., Zhang C. (2021). Data-driven multi-objective optimization of laser welding parameters of 6061-T6 aluminum alloy. J. Phys. Conf. Ser..

[15] Zhang B., Chen H., Liu Y., Meng Y., Deng A., Wu X. (2024). Study on the influence of Al-Si welding wire on porosity sensitivity in laser welding and process optimization. Opt. Laser Technol..

[16] Cui W., Li X., Zhou S., Weng J. (2007). Investigation on process parameters of electrospinning system through orthogonal experimental design. J. Appl. Polym. Sci..

[17] Ai Y., Han S., Lei C., Cheng J. (2023). The characteristics extraction of weld seam in the laser welding of dissimilar materials by different image segmentation methods. Opt. Laser Technol..

[18] Ai Y., Lei C., Cheng J., Mei J. (2023). Prediction of weld area based on image recognition and machine learning in laser oscillation welding of aluminum alloy. Opt. Lasers Eng..

[19] Hou D., Zhang W., Chen K., Lin S.-J., Yu N. (2019). Reversible Data Hiding in Color Image with Grayscale Invariance. IEEE Trans. Circuits Syst. Video Technol..

[20] Rakshit S., Ghosh A., Shankar B.U. (2007). Fast mean filtering technique (FMFT). Pattern Recognit..

[21] Mostafa M., Laifi J., Ashari M., Alrowaili Z., Criado M. (2020). MATLAB Image Treatment of Copper-Steel Laser Welding. Adv. Mater. Sci. Eng..

[22] Yang Y., Miao C., Li X., Mei X. (2014). On-line conveyor belts inspection based on machine vision. Optik.

[23] Song C., Dong S., He P., Yan S., Zhao X. (2019). Correlation of Process Parameters and Porosity in Laser Welding of 7A52 Aluminum Alloy using Response Surface Methodology. Procedia Manuf..

